# Association between dietary niacin intake and abdominal aortic calcification among the US adults: the NHANES 2013–2014

**DOI:** 10.3389/fnut.2024.1459894

**Published:** 2024-11-28

**Authors:** Jiqian Zhang, Ming Li, Xinyi Wang, Tongxin Wang, Wende Tian, Hao Xu

**Affiliations:** ^1^Graduate School, Beijing University of Chinese Medicine, Beijing, China; ^2^National Clinical Research Center for Chinese Medicine Cardiology, Xiyuan Hospital, China Academy of Chinese Medical Sciences, Beijing, China; ^3^Graduate School, China Academy of Chinese Medical Sciences, Beijing, China

**Keywords:** dietary niacin intake, abdominal aortic calcification, cardiovascular disease, cross-sectional study, NHANES

## Abstract

**Background:**

Abdominal aortic calcification (AAC) serves as a reliable predictor of future cardiovascular incidents. This study investigated the association between dietary niacin intake and AAC in US adults.

**Methods:**

In this study, we conducted a cross-sectional study of 2,238 individuals aged 40 years and older using data from the National Health and Nutrition Examination Survey (NHANES) 2013–2014. AAC was evaluated using the Kauppila scoring system through dual-energy X-ray absorptiometry. Daily niacin intake was calculated by averaging the two dietary recalls and classified in tertiles for analysis. In this study, multiple regression analyses and smoothed curve fitting were used to examine the relationship between dietary niacin intake and AAC, subgroup analyses and interaction tests were used to assess the stability of this relationship across different segments of the population, and forest plots were used to present the results. In addition, we validated the predictive performance of dietary niacin intake on the risk of severe AAC through Receiver Operating Characteristic (ROC) curve analysis.

**Results:**

Among 2,238 participants aged >40 years, the results showed that the higher dietary niacin intake group was associated with lower AAC score (*β* = −0.02, 95% CI: −0.04 – −0.01), and a lower risk of severe AAC (OR = 0.97, 95% CI: 0.96–0.99). In the fully adjusted model, the higher tertile group was associated with lower AAC score (*β* = −0.37, 95% CI: −0.73 – −0.02; *P* for trend = 0.0461) and a lower risk of severe AAC (OR = 0.60, 95% CI: 0.38–0.93; *P* for trend = 0.0234). The relationship between dietary niacin intake and AAC differed significantly between diabetic and non-diabetic population. The ROC curve analysis revealed that the area under the curve (AUC) for predicting severe AAC risk based on dietary niacin intake was 0.862, indicating good predictive performance.

**Conclusion:**

Higher dietary niacin intake group was associated with lower AAC score and a lower risk of severe AAC. Our findings suggest that dietary niacin intake has the potential to offer benefits in preventing AAC in the general population.

## 1 Introduction

Vascular calcification (VC) involves the unusual buildup of calcium phosphate crystals within the walls of blood vessels (1, 2). Notably, the abdominal aorta is the first vascular site where signs of atherosclerotic calcification appear, typically before it is observed in the coronary arteries (3, 4). Cardiovascular disease (CVD) has accounted for the largest number of deaths in the United States over the past century (5). Abdominal aortic calcification (AAC) serves as a reliable predictor of future cardiovascular incidents, proving more effective than the Framingham risk score (6). Many previous epidemiological studies have demonstrated the correlation between AAC and many cardiovascular diseases and cardiovascular deaths (6–10). Currently, there are no clinically effective treatments for AAC. Consequently, preventing the onset and progression of AAC is essential.

Niacin serves as a nutritional precursor to nicotinamide adenine dinucleotide (NAD) and nicotinamide adenine dinucleotide phosphate (NADP) (11), both of which are vital for cellular metabolic processes and energy metabolism (12). Niacin supplementation prevents atherosclerosis progression and has been shown in some studies to reduce carotid atherosclerosis and carotid intima-media thickness (cIMT) (13, 14). Results of a study suggest that therapeutic increases in NAD concentrations may protect against age-related declines in health and that dietary supplements with niacin as the primary nutritional NAD precursor may provide anti-aging properties (15). Therefore, niacin could potentially offer clinical benefits in preventing AAC. Niacin is found in a wide range of plant and animal products such as fortified grains, meats, and vegetables (12). The recommended therapeutic dose range for niacin is 1–3 g/d for dyslipidemia (in combination with statins) (16). However, few studies have investigated the association between dietary niacin intake and AAC in the general population.

The relationship between dietary niacin intake levels and AAC is not fully understood and requires further study. Therefore, we explored the relationship between dietary niacin intake and AAC based on data from the 2013–2014 NHANES.

## 2 Methods

### 2.1 Study population

Data were sourced from NHANES, which is managed by the National Center for Health Statistics (NCHS) to evaluate the health and nutritional status of the U.S. population. The sample included in NHANES exhibits a relatively high degree of representativeness due to the stratified multi-stage probability sampling methodology used in the study design. All participants provided written informed consent. The data can be accessed at https://www.cdc.gov/nchs/nhanes/ (17).

**Figure 1:**
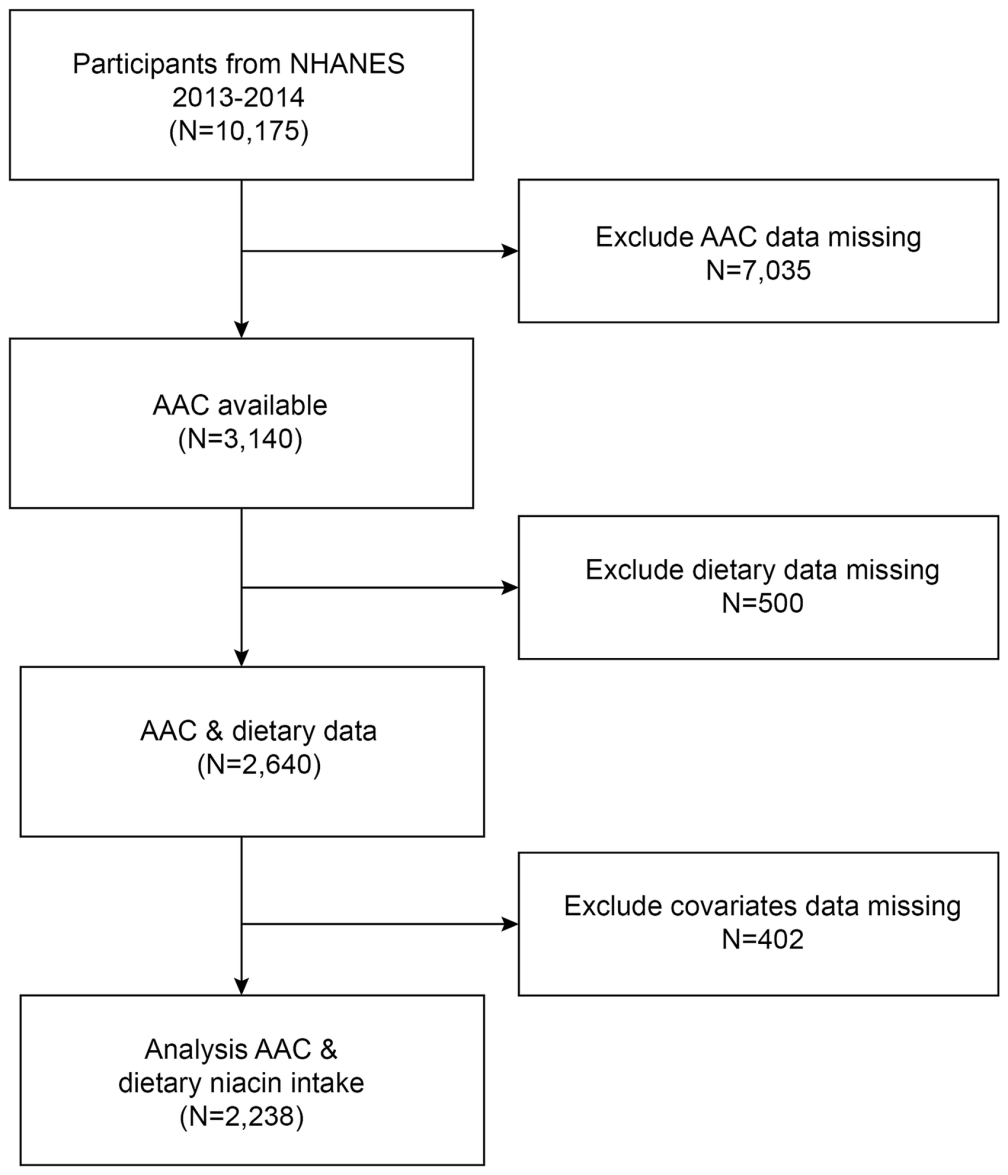
Flow chart of participants selection. NHANES, National Health and Nutrition Examination Survey.

The survey spanned a two-year period (2013–2014) within a single survey cycle. Eligibility criteria for participants included individuals aged 40 years or older, who were not pregnant, and had no reported radiation exposure in the previous 7 days. A total of 7,035 subjects with missing AAC data, 500 subjects lacking data on dietary niacin intake, and 402 subjects with incomplete covariate data were excluded. Consequently, 2,238 participants remained and were included in the analysis (Figure 1).

### 2.2 Dietary niacin intake

Dietary niacin intake was assessed using the dietary interview component, which involved interviewing participants for two 24-h dietary recalls to detail the type and amount of food consumed in the prior 24 h. For this study, daily niacin intake was calculated by averaging the two recalls.

### 2.3 AAC

Abdominal aortic calcification was evaluated using the Kauppila scoring system through dual-energy X-ray absorptiometry. The AAC score quantified the severity of calcification, with higher scores indicating more severe calcification (18). The assessment divided the abdominal aortic wall into four sections. Each section received a score from 0 to 6, based on the level of calcium deposition, leading to a cumulative AAC score ranging from 0 to 24. Typically, an AAC score > 6 is considered as severe AAC (19).

### 2.4 Covariates

Covariates in the study included gender, age, race, education level, poverty-to-income ratio (PIR), body mass index (BMI), smoking status, alcohol drinking status, diabetes, coronary heart disease, and blood biochemistry including cholesterol, creatinine, uric acid, phosphorus, calcium, and vitamin D. Smoking status was determined by whether individuals had smoked more than 100 cigarettes in their lifetime. Alcohol consumption was defined by having at least 12 alcohol drinks per year.

### 2.5 Statistical analysis

Categorical variables were reported as frequencies (percentages), while continuous variables were reported as mean ± standard error (SE). Dietary niacin intake was categorized into tertiles and baseline characteristics of participants were compared using chi-square tests and non-parametric tests. Multivariate linear regression was performed with AAC scores as a continuous variable, along with logistic regression using severe AAC as a dichotomous variable, in order to examine the relationship between dietary niacin intake and AAC. In the crude model, there were no covariates adjusted. In the model 1, covariates were adjusted for gender, age, and race. In the model 2, covariates were adjusted for gender, age, race, education level, PIR, BMI, smoking status, alcohol drinking status, cholesterol, creatinine, uric acid, phosphorus, calcium, vitamin D, diabetes, and coronary heart disease. Subgroup analyses were conducted by stratifying participants based on gender (male/female), age (below 60 years; 60 years and older), BMI (BMI less than 24.9 kg/m^2^; BMI between 25 and 29.9 kg/m^2^; BMI 30 kg/m^2^ or higher), diabetes (yes/no) and coronary heart disease (yes/no) and results were presented through forest plots. The evaluation of the non-linear relationship was conducted using the smoothed curve fitting approach. We also validated the predictive performance of dietary niacin intake on the risk of severe AAC through Receiver Operating Characteristic (ROC) curve analysis. All analyses were performed with R version 4.2 and Empower software. A two-sided *p*-value less than 0.05 is considered statistically significant.

## 3 Results

### 3.1 Baseline characteristics

This study enrolled 2,238 participants with an average age of 58.9 ± 11.9 years. The average AAC score for the overall population was 1.66 ± 3.59, with 210 (9.38%) participants diagnosed with severe AAC. The average dietary niacin intake was (24.43 ± 11.83) mg/day, with a range of tertile 1: <18.47 mg/day; tertile 2: 18.47–26.58 mg/day; tertile 3: >26.58 mg/day. Significant differences were observed in the mean AAC score among the three patient groups, with the highest niacin intake group exhibiting the lowest scores. Participants with a higher dietary niacin intake level tended to be younger, more often male, more likely to smoke and drink alcohol. They had a higher education level and belonged to a higher socioeconomic status. Additionally, they tended to have lower cholesterol and phosphorus levels, along with higher uric acid levels (Table 1).

**Table 1:** Basic characteristics of participants by dietary niacin intake among U.S. adults.

Characteristics	Dietary niacin intake				*P*-value
	T1 (*N* = 746)	T2 (*N* = 746)	T3 (*N* = 746)	All (*N* = 2,238)	
Age (years)	60.77 ± 11.94	59.30 ± 12.12	56.67 ± 11.26	58.92 ± 11.90	<0.001
Gender, *N* (%)					<0.001
Male	198 (26.54)	347 (46.51)	523 (70.11)	1,068(47.72)	
Female	548 (73.46)	399 (53.49)	223 (29.89)	1,170(52.28)	
Race, *N* (%)					0.161
Non-Hispanic White	337 (45.17)	387 (51.88)	355 (47.59)	1,079(48.21)	
Non-Hispanic Black	158 (21.18)	134 (17.96)	132 (17.69)	424(18.95)	
Mexican American	96 (12.87)	83 (11.13)	96 (12.87)	275(12.29)	
Other Hispanic	155 (20.78)	142 (19.03)	163 (21.85)	460(20.55)	
Education level, *N* (%)					0.001
<High school	175 (23.46)	121 (16.22)	131 (17.56)	427(19.08)	
High school	163 (21.85)	190 (25.47)	157 (21.05)	510(22.79)	
>High school	408 (54.69)	435 (58.31)	458 (61.39)	1,301(58.13)	
Diabetes, *N* (%)					0.057
Yes	142 (19.03)	111 (14.88)	114 (15.28)	367(16.40)	
No	604 (80.97)	635 (85.12)	632 (84.72)	1,871(83.60)	
Coronary heart disease, *N* (%)					0.769
Yes	44 (5.90)	43 (5.76)	38 (5.09)	125(5.59)	
No	702 (94.10)	703 (94.24)	708 (94.91)	2,113(94.91)	
Smoking status, *N* (%)					0.002
Yes	313 (41.96)	347 (46.51)	382 (51.21)	1,042(46.56)	
No	433 (58.04)	399 (53.49)	364 (48.79)	1,196(53.44)	
Alcohol drinking status, *N* (%)					<0.001
Yes	470 (63.00)	561 (75.20)	604 (80.97)	1,635(73.06)	
No	276 (37.00)	185 (24.80)	142 (19.03)	603(26.94)	
Family PIR	2.48 ± 1.57	2.98 ± 1.68	2.87 ± 1.63	2.78 ± 1.64	<0.001
BMI (kg/m^2^)	28.97 ± 5.75	28.42 ± 5.52	28.51 ± 5.47	28.64 ± 5.59	0.163
Cholesterol (mg/dL)	197.96 ± 43.87	192.45 ± 41.33	193.18 ± 44.10	194.53 ± 43.17	0.042
Creatinine (mg/dL)	0.95 ± 0.77	0.93 ± 0.35	0.96 ± 0.33	0.95 ± 0.52	<0.001
Uric acid (mg/dL)	5.32 ± 1.39	5.40 ± 1.39	5.57 ± 1.33	5.43 ± 1.37	<0.001
Phosphorus (mg/dL)	3.84 ± 0.58	3.81 ± 0.56	3.75 ± 0.58	3.80 ± 0.57	0.013
Calcium (mg/dL)	9.48 ± 0.40	9.46 ± 0.35	9.44 ± 0.36	9.46 ± 0.37	0.196
Vitamin D (nmol/L)	71.44 ± 31.86	73.69 ± 29.41	69.21 ± 26.17	71.45 ± 29.28	0.017
AAC score	2.05 ± 4.08	1.70 ± 3.60	1.24 ± 2.95	1.66 ± 3.59	0.001
Severe AAC, *N* (%)					<0.001
Yes	94 (12.60)	70 (9.38)	46 (6.17)	210(9.38)	
No	652 (87.40)	676 (90.62)	700 (93.83)	2,028(90.62)	

Mean ± SE for continuous variables; frequencies (percentages) for categorical variables. PIR, the ratio of income to poverty; BMI, body mass index; T, tertile; AAC, abdominal aortic calcification.

### 3.2 Association between dietary niacin intake and AAC

**Figure 2:**
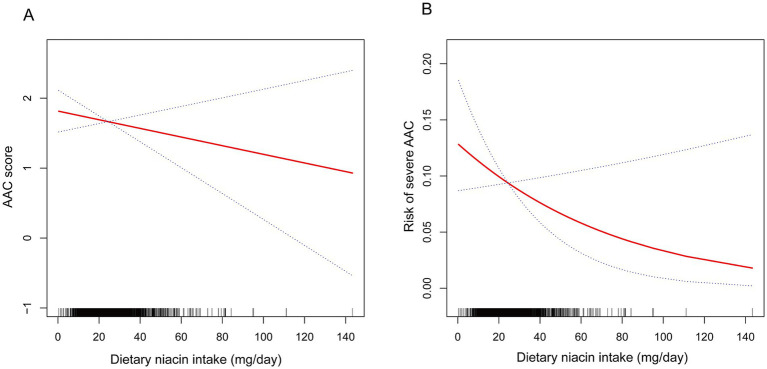
The non-linear associations between dietary niacin intake and AAC. The solid red line represents the smooth curve fit between variables. Blue bands represent the 95% confidence interval from the fit. **(A)** Dietary niacin intake and AAC score; **(B)** Dietary niacin intake and the risk of severe AAC.

Table 2 presents the association between dietary niacin intake and AAC. The crude model revealed a negative association between dietary niacin intake and AAC score (*β* = −0.02, 95% CI: −0.04 – −0.01), indicating that each increase of 1 mg/d in niacin was associated with a decrease of 0.02 units in the AAC score. However, this association was not significant in both the minimally adjusted and fully adjusted models. Additionally, a significant negative association between dietary niacin intake and severe AAC in the crude model, with each 1 mg/d increase in niacin intake associated with a 3% decrease in the risk of severe AAC (OR = 0.97, 95% CI: 0.96–0.99). After classifying dietary niacin intake into tertiles, the higher tertile group showed a decrease in AAC score (*β* = −0.37, 95% CI: −0.73- −0.02; *P* for trend = 0.0461). Participants in the higher dietary niacin intake tertile exhibited a significantly 40% reduction in the risk of severe AAC compared to those in the lower tertile (OR = 0.60, 95% CI: 0.38–0.93; *P* for trend = 0.0234). Furthermore, the non-linear negative correlation between dietary niacin intake and both AAC score and severe AAC was confirmed through smoothed curve fitting (Figure 2).

**Table 2:** Association between dietary niacin intake and AAC.

Niacin	AAC Score	Severe AAC
	*β* (95% CI), *P*-value	OR (95%CI), *P*-value
Crude model		
Continuous	−0.02 (−0.04, −0.01) 0.0003	0.97 (0.96, 0.99) 0.0003
Categories		
T1	0(ref)	1(ref)
T2	−0.35 (−0.72, 0.01) 0.0569	0.72 (0.52, 1.00) 0.0477
T3	−0.81 (−1.17, −0.45) <0.0001	0.46 (0.32, 0.66) <0.0001
*P* for trend	<0.0001	<0.0001
Minimally adjusted model (Model 1)		
Continuous	−0.01 (−0.02, 0.01) 0.2646	0.99 (0.97, 1.00) 0.1225
Categories		
T1	0(ref)	1(ref)
T2	−0.26 (−0.59, 0.08) 0.1386	0.72 (0.50, 1.04) 0.0838
T3	−0.42 (−0.78, −0.06) 0.0220	0.62 (0.41, 0.95) 0.0283
*P* for trend	0.0254	0.0281
Fully adjusted model (Model 2)		
Continuous	−0.01 (−0.02, 0.01) 0.3242	0.99 (0.97, 1.00) 0.1135
Categories		
T1	0(ref)	1(ref)
T2	−0.22 (−0.56, 0.11) 0.1884	0.69 (0.47, 1.01) 0.0573
T3	−0.37 (−0.73, −0.02) 0.0407	0.60 (0.38, 0.93) 0.0234
*P* for trend	0.0461	0.0244

Crude model: no covariates were adjusted. Model 1: gender, age, and race were adjusted. Model 2: gender, age, race, education level, PIR, BMI, smoking status, alcohol drinking status, cholesterol, creatinine, uric acid, phosphorus, calcium, vitamin D, diabetes, and coronary heart disease were adjusted. PIR, the ratio of income to poverty; BMI, body mass index; T, tertile; AAC, abdominal aortic calcification.

### 3.3 Subgroup analyses

**Figure 3:**
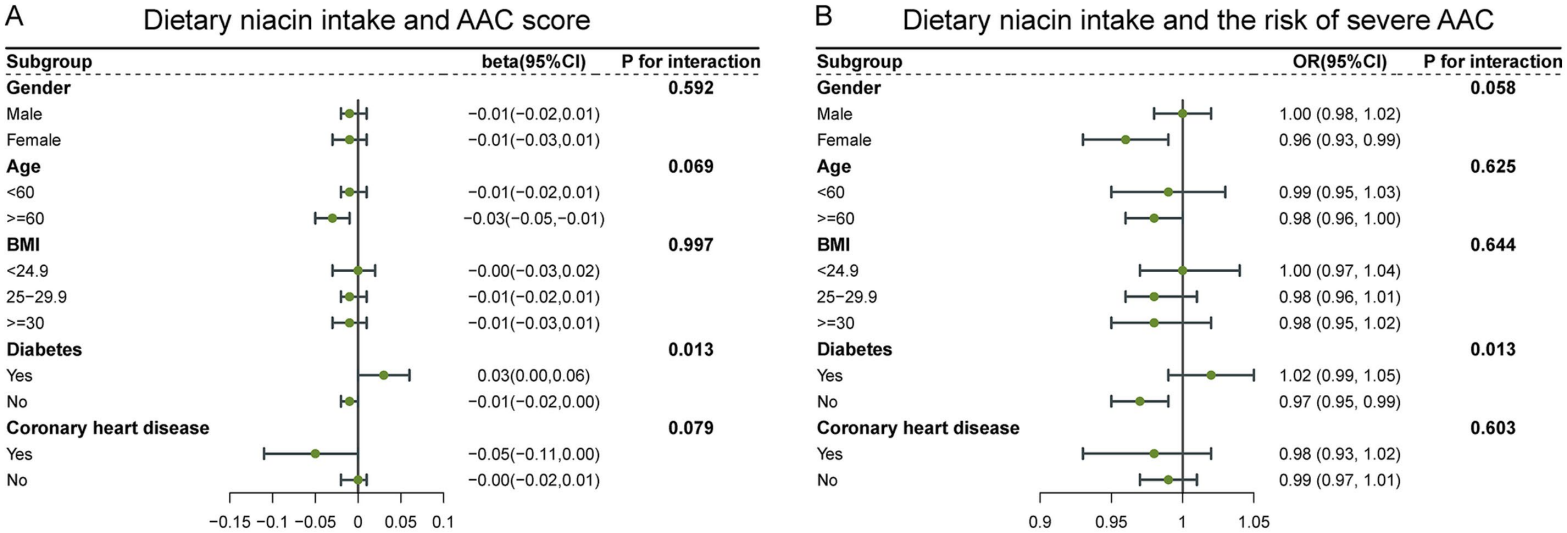
Forest plots for subgroup analysis of dietary niacin intake and AAC. **(A)** Dietary niacin intake and AAC score; **(B)** Dietary niacin intake and the risk of severe AAC.

To evaluate the stability of the relationship between dietary niacin intake and AAC across the general population and to identify variations in different demographic settings, we conducted subgroup analyses and interaction tests stratified by age, gender, BMI, diabetes, and coronary heart disease (Tables 3, 4) and presented the results through forest plots (Figure 3). The relationship between dietary niacin intake and both AAC score and severe AAC showed significant differences between diabetic and non-diabetic populations (*P* for interaction = 0.013). Additionally, no statistically significant relationships were observed in other subgroup analyses.

**Table 3:** Subgroup analysis of the association between dietary niacin intake and AAC score.

Subgroup	AAC score [*β* (95%CI)]	*P* for interaction
Gender		0.592
Male	−0.01 (−0.02, 0.01)	
Female	−0.01 (−0.03, 0.01)	
Age		0.069
<60 years	−0.01 (−0.02, 0.01)	
≥60 years	−0.03 (−0.05, −0.01)	
BMI		0.997
<24.9 kg/m^2^	−0.00 (−0.03, 0.02)	
25–29.9 kg/m^2^	−0.01 (−0.02, 0.01)	
≥30 kg/m^2^	−0.01 (−0.03, 0.01)	
Diabetes		0.013
Yes	0.03 (0.00, 0.06)	
No	−0.01 (−0.02, 0.00)	
Coronary heart disease		0.079
Yes	−0.05 (−0.11, 0.00)	
No	−0.00 (−0.02, 0.01)	

Gender, age, race, education level, PIR, BMI, smoking status, alcohol drinking status, cholesterol, creatinine, uric acid, phosphorus, calcium, vitamin D, diabetes, and coronary heart disease were adjusted. PIR, the ratio of income to poverty; BMI, body mass index; AAC, abdominal aortic calcification.

**Table 4:** Subgroup analysis of the association between dietary niacin intake and severe AAC.

Subgroup	Severe AAC [OR (95%CI)]	*P* for interaction
Gender		0.058
Male	1.00 (0.98, 1.02)	
Female	0.96 (0.93, 0.99)	
Age		0.625
<60 years	0.99 (0.95, 1.03)	
≥60 years	0.98 (0.96, 1.00)	
BMI		0.644
<24.9 kg/m^2^	1.00 (0.97, 1.04)	
25–29.9 kg/m^2^	0.98 (0.96, 1.01)	
≥30 kg/m^2^	0.98 (0.95, 1.02)	
Diabetes		0.013
Yes	1.02 (0.99, 1.05)	
No	0.97 (0.95, 0.99)	
Coronary heart disease		0.603
Yes	0.98 (0.93, 1.02)	
No	0.99 (0.97, 1.01)	

Gender, age, race, education level, PIR, BMI, smoking status, alcohol drinking status, cholesterol, creatinine, uric acid, phosphorus, calcium, vitamin D, diabetes, and coronary heart disease were adjusted. PIR, the ratio of income to poverty; BMI, body mass index; AAC, abdominal aortic calcification.

### 3.4 Predictive ability

**Figure 4:**
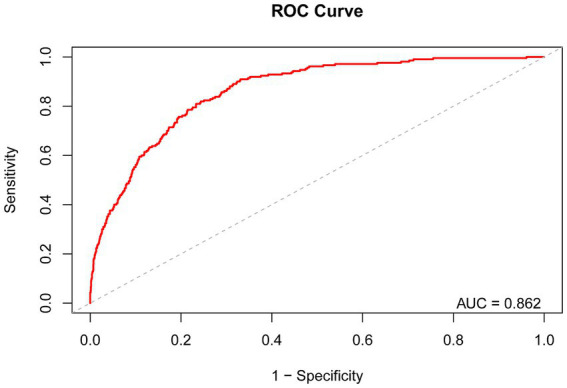
Receiver Operating Characteristic curve and AUC value of dietary niacin intake for predicting the risk of severe AAC.

We validated the predictive performance of dietary niacin intake on the risk of severe AAC through Receiver Operating Characteristic (ROC) curve analysis. The ROC curve analysis revealed that the area under the curve (AUC) for predicting severe AAC risk based on dietary niacin intake was 0.862 (Figure 4). The study indicates that that dietary niacin intake demonstrates good performance in predicting the risk of severe AAC.

## 4 Discussion

To our knowledge, this study may be the first to explore the relationship between dietary niacin intake and AAC. Our results indicate that participants in the higher dietary niacin intake group exhibited lower AAC scores and a reduced risk of severe AAC compared to those in the lower dietary niacin intake group. Increased niacin intake in non-diabetics can significantly help reduce the risk of AAC, however this association does not appear to be consistent in the diabetic population. In addition, dietary niacin intake showed good performance in predicting the risk of severe AAC.

To date, no studies have specifically explored the relationship between dietary niacin intake and AAC, although existing research has highlighted the impact of dietary factors on AAC. Our earlier studies found that dietary vitamin C intake was inversely related to both the risk of AAC and AAC score (20), and higher HEI-2015 scores were associated with lower AAC scores and a decreased risk of developing AAC (21). Other studies revealed that higher dietary selenium intake, dietary live microbe intake, and dietary copper intake all exhibited negative correlations with lower risk of severe AAC (22–24). In addition, it has been shown that niacin supplementation can alleviate vascular calcification by reducing dietary phosphate absorption (25, 26). A single-center randomized controlled study also showed that nicotinamide, a metabolite of niacin, not only reduces the blood phosphorus level in chronic hemodialysis patients but also decreases the fibroblast growth factor 23 (FGF 23) level and slows down the rate of decline of Klotho, which in turn exerts a protective effect against vascular calcification (27). However, a randomized controlled trial showed that the addition of niacin to statin therapy failed to provide protection against major adverse cardiovascular events (MACE) in patients with cardiovascular disease, despite significantly improving HDL cholesterol and triglyceride levels (28). Another study also showed that the terminal metabolites of excess niacin, especially N1-methyl-4-pyridone3-carboxamide (4PY), were associated with incident MACE risks (29). Niacin has shown some potential in improving specific metabolic markers and chronic diseases, but its role in the prevention and treatment of CVD remains complex and uncertain. This calls for continued in-depth exploration of the mechanisms and long-term effects of niacin and its metabolites in CVD.

The observed association between dietary niacin intake and AAC may be influenced by a variety of factors. Vascular calcification is mainly controlled by vascular smooth muscle cells (VSMCs), and the main factors identified to drive VSMCs differentiation include oxidative stress, inflammation, aging, and uremia (30). Oxidative stress causes VSMCs to produce more reactive oxygen species (ROS), which promotes the expression of the osteogenic transcription factor Runx2, resulting in the transition of VSMCs from a contractile phenotype to an osteogenic phenotype (31, 32). One study showed that increased ROS production which is mainly located around calcified lesions enhances the progression of aortic valve calcification (33). Inflammatory cells, especially macrophages/monocytes, as well as pro-inflammatory factors can also promote VSMCs differentiation (34, 35).

Niacin and its major metabolite nicotinamide significantly inhibit monocyte/macrophage adhesion and accumulation (36), and also exert antioxidant and anti-inflammatory properties by reducing the production of ROS, NO, and pro-inflammatory cytokines, in activated human mature macrophages (37). It has been shown that niacin/nicotinamide restriction increases ROS and supplementation with niacin/nicotinamide completely reverses ROS accumulation (38) and protects them from oxidative stress damage (39). In addition, niacin reduced pro-inflammatory cytokine levels in oxLDL-treated human THP-1 macrophages (40), LPS-treated mouse alveolar macrophages (41), and bone marrow-derived macrophages (42). Aging is also a driver of VSMCs differentiation, and significant reductions in NAD levels are also associated with aging; restoring NAD levels prevents age-related health decline (15, 43), and niacin significantly restores muscle and whole-body NAD (44).

Our study found significant differences in the association of dietary niacin intake with AAC score and severe AAC between diabetic and non-diabetic populations. It has been observed in clinical trials that niacin may increase blood glucose levels in diabetic patients (45, 46) and increase the prevalence of diabetes (47). A meta-analysis that included 11 trials totaling 26,340 subjects without diabetes showed that niacin treatment increased the risk of developing diabetes by 34% (48). Another retrospective study that included 1,112 subjects found that the risk of developing coronary artery calcium was significantly associated with elevated fasting plasma glucose (FPG) (49). Considering the impact of niacin on patients with diabetes and its influence on abdominal aortic calcification, it appears that higher niacin intake might be more strongly associated with a reduced risk of developing AAC, although this association does not appear to be consistent within the diabetic population.

The primary strengths of our study include the use of NHANES data, which employs a multistage sampling methodology to enhance the reliability and robustness of the findings. Additionally, the representative nature of the population sample allows the findings to be applicable to the adult population across the United States. Furthermore, this is the first study to explore the relationship between dietary niacin intake and AAC, providing valuable insights into how diet impacts AAC. However, several limitations must be acknowledged. Primarily, while we can conclude that dietary niacin intake correlates with AAC, we cannot establish a causal relationship between them. Despite accounting for multiple covariates, we could not eliminate the influence of all potential confounders. Additionally, self-reported dietary intake data might be susceptible to recall bias and estimation errors. Finally, because the NHANES participants were all from the U.S. population, it is unclear whether the findings are applicable to populations from other regions, and further validation is needed.

## 5 Conclusion

Higher dietary niacin intake group was associated with lower AAC score and a lower risk of severe AAC. Dietary niacin intake has the potential to offer benefits in preventing AAC in the general population.

## Data Availability

The raw data supporting the conclusions of this article will be made available by the authors, without undue reservation.
